# Lipid-lowering pharmacotherapy and socioeconomic status: atherosclerosis risk in communities (ARIC) surveillance study

**DOI:** 10.1186/1471-2458-13-488

**Published:** 2013-05-20

**Authors:** Joseph P Kitzmiller, Randi E Foraker, Kathy M Rose

**Affiliations:** 1 Departments of Pharmacology and Biomedical Engineering, The Ohio State University Medical Center, 5072B Graves Hall 333 West 10th Avenue, Columbus, OH, 43210, USA; 2 Division of Epidemiology, College of Public Health, The Ohio State University, 334 Cunz Hall, 1841 Neil Avenue, Columbus, OH, 43230, USA; 3 Department of Epidemiology, Gillings School of Global Public Health, University of North Carolina, 135 Dauer Drive, Chapel Hill, NC, 27599, USA; 4 SRA International, Inc, 2605 Meridian Parkway, Durham, NC, 27713, USA

**Keywords:** Lipids, Socioeconomics, Statins, Lipid-lowering pharmacotherapy, Cardiovascular disease

## Abstract

**Background:**

Lipid-reduction pharmacotherapy is often employed to reduce morbidity and mortality risk for patients with dyslipidemia or established cardiovascular disease. Associations between socioeconomic factors and the prescribing and use of lipid-lowering agents have been reported in several developed countries.

**Methods:**

We evaluated the association of census tract-level neighborhood household income (nINC) and lipid-lowering medications received during hospitalization or at discharge among 3,546 (5,335 weighted) myocardial infarction (MI) events in the United States (US) Atherosclerosis Risk In Communities (ARIC) surveillance study (1999–2002). Models included neighborhood household income, race, gender, age, study community, year of MI, hospital type (teaching vs. nonteaching), current or past history of hypertension, diabetes or heart failure, and presence of cardiac pain.

**Results:**

About fifty-nine percent of patients received lipid-lowering pharmacotherapy during hospitalization or at discharge. Low nINC was associated with a lower likelihood (prevalence ratio 0.89, 95% confidence interval: 0.79, 1.01) of receiving lipid-lowering pharmacotherapy compared to high neighborhood household income, and no significant change in this association resulted when adjusted for the above-mentioned covariates.

**Conclusion:**

Patient’s socioeconomic status appeared to influence whether they were prescribed a lipid-lowering pharmacotherapy after hospitalization for myocardial infarction in the US ARIC surveillance study (1999–2002).

## Background

Lipid reduction can significantly improve cardiovascular risk and lower morbidity and mortality following myocardial infarction (MI). Pharmacotherapy, along with lifestyle changes, plays an essential role in improving lipid profiles. Statins, arguably the most efficacious of the lipid-lowering drug classes, are often first-line therapy for the dyslipidemias and for decreasing cardiovascular risk. However, investigators worldwide have reported that socioeconomic factors often influence the prescribing and use of statins and other lipid-lowering agents. In 2007 Ward et. al. published data from four primary-care trusts in Northwest England that indicated ethnic inequities in statin prescribing rates [1]. A Danish study published in 2005 reported that among men with cardiovascular disease, statin use was higher in those with the highest socioeconomic status (SES) and lower among retired men in old-age pensioners compared to basic-level workers [2]. An Australian study published in 2004 found that statins were prescribed for males when indicated more often to those with higher SES and were prescribed for females at higher rates at lower levels of risk [3]. In 2006 a report describing significant disparities in the use of lipid-lowering agents in the United States (US) was published [4]; however, such studies investigating SES and the use of lipid-lowering medications in the US are rare. Our previous work described differential receipt of aspirin, beta-blockers, and angiotensin converting enzyme inhibitors by neighborhood SES [5]. This brief report discusses our subsequent investigation into the relationship between neighborhood SES and lipid-lowering pharmacotherapy.

## Methods

We evaluated the association between tertiles (low, medium, and high) of census tract-level neighborhood household income (nINC) and lipid-lowering medications received during hospitalization or at discharge among 3,546 (5,335 weighted) MI events in the US Atherosclerosis Risk In Communities (ARIC) surveillance study (1999–2002). The ARIC study’s community-based surveillance of coronary heart disease has been ongoing since 1987 and is designed to capture MI and fatal coronary heart disease events in four US communities [Jackson, Mississippi (MS); Forsyth County, North Carolina (NC); Washington County, Maryland (MD); and Minneapolis, Minnesota (MN)]). While it comprises the same communities from which ARIC cohort members were recruited, ARIC community surveillance does not include in-person physical exams, annual follow-up, or any contact with ARIC cohort participants (unless they happen to be sampled as a surveillance case). ARIC community-surveillance staff ascertained coronary heart disease-related hospital discharges and deaths and abstracted data related to the event of interest. Institutional Review Board (IRB) approvals were obtained by each participating ARIC study center (the Universities of NC, MS, MN, and John Hopkins University) and the coordinating center (University of NC), and the research was conducted in accordance with the principles described in the Declaration of Helsinki. Data for this study were abstracted from medical records and strict data confidentiality was maintained. Further details regarding ARIC’s methods for data collection are provided elsewhere [6]. For our analyses, we weighted the hospitalized MI cases based on the probability sampling of selected International Classification of Disease codes [6] in order to estimate the eligible population of cases that would have been studied had the probability sampling not been employed.

We estimated prevalence ratios and 95% confidence intervals for receipt of lipid-lowering medication post-MI using weighted Poisson regression, and we used generalized estimation equations (PROC GENMOD, SAS Institute) to account for the clustering of MI events within census tracts and within patients, as incident and recurrent MI events were considered together based upon our previous analyses [7,8]. Model 1 included nINC, race, gender, age, study community and year of MI. Model 2 included the same parameters as well as hospital type (teaching vs. nonteaching), current or past history of hypertension, diabetes or heart failure, and presence of cardiac pain. Additionally, we examined whether the following parameters modified the nINC – lipid-lowering therapy relationship: race, gender, age, study community and year of MI. Models utilizing tertiles defined by overall nINC cut-points were evaluated, as interpretations of our earlier work in this population did not change based on the delineation (community-specific, race-specific, and overall cut-points) of nINC tertiles [9].

## Results and discussion

**Figure 1:**
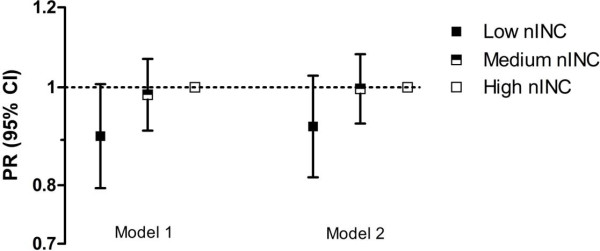
**Receipt of lipid-lowering pharmacotherapy among Atherosclerosis Risk in Community (ARIC) surveillance patients (1999–2002).** Model 1: Neighborhood income (nINC), race, gender, age, study community, year of myocardial infarction (MI). Model 2: Model 1 plus hospital type (teaching vs. nonteaching), current or past history of hypertension, diabetes or heart failure, and presence of cardiac pain.

The MI cases were distributed across the four ARIC study communities as follows: 25.7% in MS, 36.7% in NC, 15.9% in MD, and 21.7% in MN. The MI patients were 34.5% female, 25.3% black, and had an average age of 60.2 years. The mean nINC was approximately $42,700. Overall, 58.5% of patients received lipid-lowering pharmacotherapy during hospitalization or at discharge. In models adjusted for race, gender, age, study community and year of MI, there was no significant effect modification of the nINC- lipid-lowering therapy relationship by race, gender, age, study community or year of MI as indicated in these data. Results of models utilizing overall cut-points are reported in Table 1 and Figure 1. In Model 1, low nINC was associated with a lower likelihood (prevalence ratio 0.89, 95% confidence interval: 0.79, 1.01) of receiving lipid-lowering pharmacotherapy compared to high nINC. This relationship did not change substantially when the additional parameters (hospital type, current or past history of hypertension, diabetes or heart failure, and presence of cardiac pain) were included (Model 2). Although lipid-lowering pharmacotherapy remains a central component of cardiovascular-risk-profile optimization, several investigators worldwide have reported significant associations among socioeconomic parameters and statin prescribing. Our investigation suggests that prescribing of lipid-lowering pharmacotherapies may be influenced by neighborhood SES in the US ARIC study population (1999–2002).

**Table 1:** Receipt of lipid-lowering pharmacotherapy among Atherosclerosis Risk in Community (ARIC) surveillance patients (1999–2002): results from regression models for selected variables

**Variable**	**Prevalence ratio**	**95% CI**
Model 1		
nINC		
Low nINC	0.89	0.79, 1.01
Medium nINC	0.98	0.91, 1.07
High nINC	1.00	ref
Gender		
Female	0.91	0.84, 0.98
Male	1.00	ref
Race		
Black	0.86	0.76, 0.97
White	1.00	ref
Model 2		
nINC		
Low nINC	0.91	0.81, 1.03
Medium nINC	1.00	0.92,1.08
High nINC	1.00	ref
Gender		
Female	0.92	0.85, 1.00
Male	1.00	ref
Race		
Black	0.90	0.79, 1.02
White	1.00	ref
Hospital type		
Teaching	0.87	0.79, 0.96
Non-teaching	1.00	ref
Medical history		
Hypertension	0.99	0.92, 1.06
Diabetes	1.05	0.97, 1.14
Heart failure	0.72	0.65, 0.79

## Conclusions

Our results demonstrate that a patient’s SES may influence whether they were prescribed a lipid-lowering pharmacotherapy after hospitalization for MI in the US ARIC surveillance study (1999–2002). This observation should be noted by clinicians, patients, and policy makers. Efforts to decrease or eliminate the influence of socioeconomic factors on the prescribing of pharmacotherapy should be implemented.

## Abbreviations

US: United States; ARIC: Atherosclerosis risk in communities; MI: Myocardial infarction; SES: Socioeconomic status; nINC: neighborhood household income; MS: Mississippi; NC: North Carolina; MD: Maryland; MN: Minneapolis

## Competing interests

The authors declare that they have no competing interests.

## Authors’ contributions

JPK drafted the manuscript and contributed to the analysis and interpretation of data. REF participated in the design of the study, acquired the data, performed the data analysis and interpretation, and assisted in drafting and revising the manuscript. KMR participated in the design of the study, assisted in the data analysis and interpretation, and also assisted in revising the manuscript. All three authors certify that they have participated sufficiently in the work and believe in its overall validity and take public responsibility for its content. All authors read and approved the final manuscript.

## Pre-publication history

The pre-publication history for this paper can be accessed here:

http://www.biomedcentral.com/1471-2458/13/488/prepub
